# Vocabulary Size in Speech May Be an Early Indicator of Cognitive Impairment

**DOI:** 10.1371/journal.pone.0155195

**Published:** 2016-05-13

**Authors:** Eiji Aramaki, Shuko Shikata, Mai Miyabe, Ayae Kinoshita

**Affiliations:** 1 Nara Institute of Science and Technology (NAIST), 8916–5 Takayama, Ikoma City, 630–0192, Japan; 2 Wakayama University, Sakaedani 930, Wakayama City, 640–8510, Japan; 3 Kyoto University Graduate School of Medicine, 54 Kawahara-cho, Syogoin, Sakyo-ku, Kyoto City, 606–8507, Japan; University of California, San Francisco, UNITED STATES

## Abstract

Little is known about the relationship between mild cognitive impairment (MCI) and changes to language abilities. Here, we used the revised Hasegawa Dementia Scale (HDS-R) to identify suspected MCI in elderly individuals. We then analyzed written and spoken narratives to compare the language abilities between study participants with and without MCI in order to explore the relationship between cognitive and language abilities, and to identify a possible indicator for the early detection of MCI and dementia. We recruited 22 people aged 74 to 86 years (mean: 78.32 years; standard deviation: 3.36). The participants were requested to write and talk about one of the happiest events in their lives. Based on HDS-R scores, we divided the participants into 2 groups: the MCI Group comprised 8 participants with a score of 26 or lower, while the Healthy Group comprised 14 participants with a score of 27 or higher. The transcriptions of both written and spoken samples for each participant were used in the measurement of NLP-based language ability scores. Our analysis showed no significant differences in writing abilities between the 2 groups in any of the language ability scores. However, analysis of the spoken narrative showed that the MCI Group had a significantly larger vocabulary size. In addition, analysis of a metric that signified the gap in content between the spoken and written narratives also revealed a larger vocabulary size in the MCI Group. Individuals with early-stage MCI may be engaging in behavior to conceal their deteriorating cognition, thereby leading to a temporary increase in their active spoken vocabulary. These results indicate the possible detection of early stages of reduced cognition before dementia onset through the analysis of spoken narratives.

## Introduction

Among other factors, increasing life expectancies have resulted in the rise of dementia as one of the most serious health and social problems in Japan. As the number of patients with dementia increases, the needs of these individuals may eventually exceed the current capacity of the national healthcare system. Data from Japan’s Ministry of Health, Labour and Welfare indicate that more than 1 in 4 elderly individuals would soon suffer from mild cognitive impairment (MCI) or dementia [1]. In addition to advancements in medical treatments, it is also important to develop methods to detect the early stages of dementia in order to prevent further deterioration and alleviate requirements for care. Although treatments should ideally be initiated prior to the onset of severe dementia symptoms, there is currently no single neuroimaging factor or biomarker that can accurately predict the progression of MCI to dementia [2–4]. Recent studies on early detection methods (such as blood testing and detailed memory testing) have revealed vast improvements in detection capabilities [5–8]. However, most of these methods are physically and/or mentally invasive, which has led to a demand for low or even non-invasive detection methods.

Dementia symptoms include degenerative cognitive decline, as well as behavioral and functional disorders. The disease also results in the deterioration of various executive functions, reasoning, and language abilities. Among these, language deficits have been shown to be more apparent from the early stages of dementia [9]. These deficits include naming disorders, auditory and written comprehension impairment, fluent but empty speech, and semantic paraphasia; however, repetition abilities and articulation are often preserved [10–15]. It has been reported that the impairment of language abilities in dementia patients is often inconsistent, as semantic and pragmatic language abilities are likely to become more impaired, whereas syntax and phonology demonstrate better preservation [16, 17].

Semantic errors have been reported to be the most common and distinct language deficit, as dementia patients tend to substitute target names with superordinate category names or demonstrate circumlocutory speech with impaired naming [13]. In addition, other studies have also reported unrelated errors [18], phonological errors [19], and visual errors [19]. However, these are often dependent on the type of picture confrontation naming task, the severity or stage of the disease, or other unique patient-level circumstances [20]. MCI, part of which constitutes a pre-stage of dementia, may indicate the boundary between aging-related non-dementia reduction in cognition and dementia on the spectrum of cognitive function. Previous studies [21] have reported conflicting results in the language abilities of MCI patients, with observations of preserved syntactic reasoning with diminished verbal fluency, impaired confrontation naming, and reduced language comprehension. Productive and receptive discourse-level processing has also been reported to be altered in patients with MCI and the early stages of Alzheimer’s disease (AD) dementia [21]. These inconsistent findings may be due to variations in methodological or diagnostic approaches [21].

There is therefore a need for further investigation into the relationship between MCI and language ability. In particular, this study focuses on the comparative relationship between oral and written narratives, which has not received sufficient attention in previous studies. We first examined if there were differences in each narrative type for differing cognitive levels. If differences were found, we then identified factors with the most influence on these differences. In this study, we employed Natural Language Processing (NLP) techniques to examine this association. Recent NLP technologies have enabled the automatic aggregation of narrative data that contain enormous amounts of lexical information for various diseases, such as frontotemporal lobar degeneration [22], autism spectrum disorder [23], and primary progressive aphasia [24]. Here, we focus on MCI in order to further understand the nature of language impairment in this condition, and to possibly identify sensitive measures of linguistic impairment that may constitute a supplementary clinical tool for the detection of MCI.

In this study, we used the revised Hasegawa Dementia Scale (HDS-R) to identify elderly individuals with suspected MCI. In an analysis of written and spoken narratives, we compared language ability scores between study participants with and without MCI (i.e., high and low HDS-R groups, respectively) in order to explore the relationship between cognitive ability and language ability, and to identify a possible indicator for the early detection of MCI and dementia.

## Methods

### Ethics Statement

The study was conducted using a publicly available database. The data, which comprised both recordings and written interviews of elderly individuals, were collected by the Silver Human Resources Center in Tokyo. The use of these data for research purposes was approved by the National Silver Human Resources Center Association Committee in accordance with the Japanese National Labour Law. The data contained no personally identifiable information, and written informed consent (including the waiver of copyrights) was obtained from all participants before analysis.

### Data Source

#### Participants

The data source of this study was the "Japanese Elder’s Language Index Corpus", or JELiCo (https://dx.doi.org/10.6084/m9.figshare.2082706.v1), which is a corpus database managed by the MedNLP Laboratory, Kyoto University, Japan. This corpus was compiled using data from 22 people aged 74 to 86 years (mean age: 78.32 years; standard deviation [SD]: 3.36) who agreed to provide data for research purposes. All except 1 of the participants had been educated at the high school level and above. The characteristics of the participants are shown in Table 1.

**Table 1:** Participant characteristics

	Healthy	MCI
	(HDS-R score of 27 or higher)	(HDS-R score of 26 or lower)
**Gender**	Men: 7; Women: 7	Men: 4; Women: 3
**Education Level**	University or above: 6; High School or above: 14	University or above: 5; High School or above: 7
**Mean Age, years (SD)**	77.21 (2.11)	80.25 (4.18)

HDS-R, revised Hasegawa Dementia Scale; MCI, mild cognitive impairment; SD, standard deviation

#### Written Narrative

The participants were requested to write about one of the happiest events in their lives. The writing assignment was distributed a day before the oral interview with instructions as shown in Table 2. The participants wrote their responses on the provided A4-size assignment sheets, which were later re-typed by one of the authors to produce the digital script for analysis. The average word count (in Japanese characters) was 497.36 characters, and ranged from 210 characters to 958 characters.

**Table 2:** Writing assignment instructions distributed to the participants.

Writing Assignment Instructions	Thank you for participating in our interview. Before your oral interview, we would like you to take part in a written assignment. Please refer to the following instructions, and bring the completed assignment to the interview site.
Procedure	We would like you to write about one of the happiest events in your life. Please use the provided manuscript papers to write your answer. If possible, the essay should be 500 characters or more. *NOTE: Please do NOT get any help from others, such as your family, when writing your assignment. The length of the assignment is just a reference. You may write more if you can or write less if it is difficult to reach the target length, but please try to work within your comfort zone. There are 200 squares on each provided manuscript paper. When you fill in 2.5 pages, you have written approximately 500 characters. For this assignment, you do not need to focus on writing eloquently. Please write freely in a style that is most comfortable to you.
Schedule	Please complete this assignment the day before the oral interview. There is no need to prepare for this assignment.

#### Spoken Narrative

The participants were requested to talk about the same theme that they had written about the day before, i.e., one of the happiest events in their lives. Their speeches were recorded using a digital recorder (all data were recorded as 48-kHz, 24-bit stereo WAV files), and a phonetic transcription of the speeches was prepared by one of the authors. The average recording time for each interview was 91.45 seconds, and ranged from 9 seconds to 248 seconds. After phonetic transcription, the average word count (in Japanese characters) was 417.73 characters, and ranged from 64 characters to 1,368 characters.

#### Measuring Cognitive Function Levels

The HDS-R is a screening test for dementia patients used in Japan that is similar to the Mini-Mental State Examination; both these tests show a high level of correlation [25]. HDS-R scores that are 20 or lower (from a possible maximum score of 30) are considered indicative of dementia (sensitivity 0.90, specificity 0.82). Normal cognitive function is indicated by HDS-R scores of 27 or higher.

We divided the 22 participants into 2 groups according to their HDS-R scores: the Low HDS-R Group comprised participants with a score of 26 or lower, indicating non-severe levels of cognitive impairment (n = 8; minimum score 22, maximum score: 26); the Healthy (high HDS-R) Group comprised participants with a score of 27 or higher, indicating normal cognitive function (n = 14; minimum score: 27, maximum score: 30). The participants’ age and gender distributions for each group are shown in Table 1.

All writing samples, speech samples, and cognitive level measurements were collected between May 13, 2015 and June 2, 2015.

### Language Ability Scores

The transcriptions of both written and spoken samples for each participant were used in the measurement of 5 NLP-based language ability scores, which are briefly summarized in Table 3. Note that the scores do not directly correspond to overall language abilities. The following indicators were used in this study:

**Table 3:** Language ability indicators.

Indicator	Indication	Description
**TTR (Type-Token Ratio)**	Vocabulary Size	Number of types / Number of tokens
**PVS (Potential Vocabulary Size)**	Vocabulary Size	Estimated vocabulary size
**VL (Vocabulary Level)**	Vocabulary Level	Ratio of intermediate-level nouns
**DepD (Dependency Distance)**	Grammatical complexity	Average gaps in each dependency arc
**Yngve Score**	Grammatical complexity	Number of average cases of a verb

#### (1) Type-Token Ratio

The Type-Token Ratio (TTR) indicates the size of an individual’s vocabulary, and is derived from the ratio of word types to tokens (i.e., type/token, where type refers to the number of different words used in a text and token refers to the number of words in a text). Orthographic variants were disambiguated using the Japanese morphological analysis system, or JUMAN [26]. In this study, we considered both the function and content of words.

$$TTR=\frac{No.\;of\;Types}{No.\;of\;Tokens}$$

#### (2) Potential Vocabulary Size

The Potential Vocabulary Size (PVS) score is a modified version of the TTR, and as such, also corresponds to an individual’s vocabulary size. As sampling size can introduce bias into the TTR, numerous variations have been proposed to replace the conventional indicator; these include Brunét’s Index [27], Honore’s Statistic [27], Moving Average TTR [28], Guiraud’s R [29], Maas’s α^2^ [30], Dugast’s K [31], Yule’s K [32], and the measure of textual lexical diversity [33]. This study analyzed the PVS score of each individual, which was calculated using the extrapolation of the number of types in their narrative samples. PVS was estimated based on Zipf’s law, which states that the frequency of a word is a power function of its rank within a text. We calculated the frequency of each word of a specific rank (k) in a population of words (N) using the following equation:

$$f(k,N)=\frac{\left(1/k\right)}{\sum_{n=1\ldots N}\left(1/n\right)}$$

Using a person with a narrative of 3 words as an example, the probability of the appearance of each word is calculated as follows: the probability of appearance of the first word is calculated as 1/ (1+1/2+1/3) = 0.55, the probability of appearance of the second word is calculated as (1/2)/ (1+1/2+1/3) = 0.27, and the probability of appearance of the third word is calculated as (1/3)/ (1+1/2+1/3) = 0.18.

**Fig 1:**
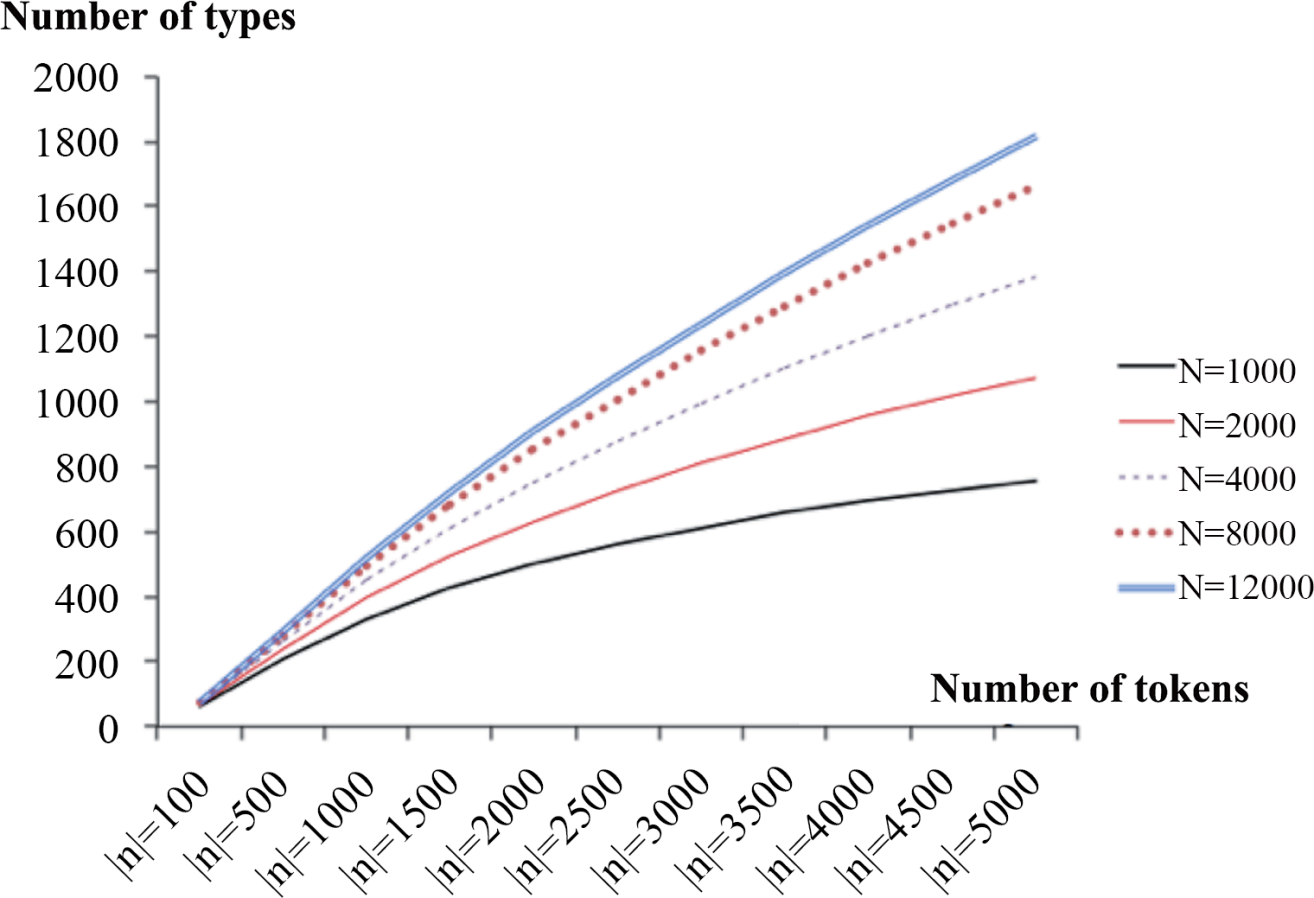
Expected type-token curve. The figure indicates the potential active vocabulary size (number of types) (*y-axis*) plotted against token sample size (*x-axis*).

In this way, we can ascertain the probability of word appearance for all words in a sample (N) beginning from rank 1 (f[1,N]) to rank N (f[N,N]). From this probability, we can estimate the relationship between the TTR and the PVS, which is presented in Fig 1. For more details, please refer to the online material (https://dx.doi.org/10.6084/m9.figshare.2082709.v1).

#### (3) Vocabulary Level

Vocabulary Level (VL) represents an individual’s average difficulty in lexical choices. This metric was originally created as a linguistic measure for non-native speakers of Japanese. Word scores were obtained from the *Japanese Learner's Dictionary* [34], where the most common 17,928 Japanese words are classified into the beginner, intermediate, and advanced levels. To derive the VL score of each participant, we extracted and totaled the number of intermediate- and higher-level nouns, and divided this figure by the total number of nouns. Word level is often quantitated by simply calculating the average levels of the participants. However, in the case of daily communication, conversations are mainly conducted using nouns at the beginner and intermediate levels. Therefore, this study focused on the ratio of intermediate words. As described in our previous study [35], Japanese language VLs are best represented using nouns; as a result, we took only nouns into account when analyzing VLs in this study.

$$VL=\frac{No.\;of\;intermediate\;or\;higher\;level\;nouns}{No.\;of\;nouns}$$

#### (4) Dependency Distance

**Fig 2:**
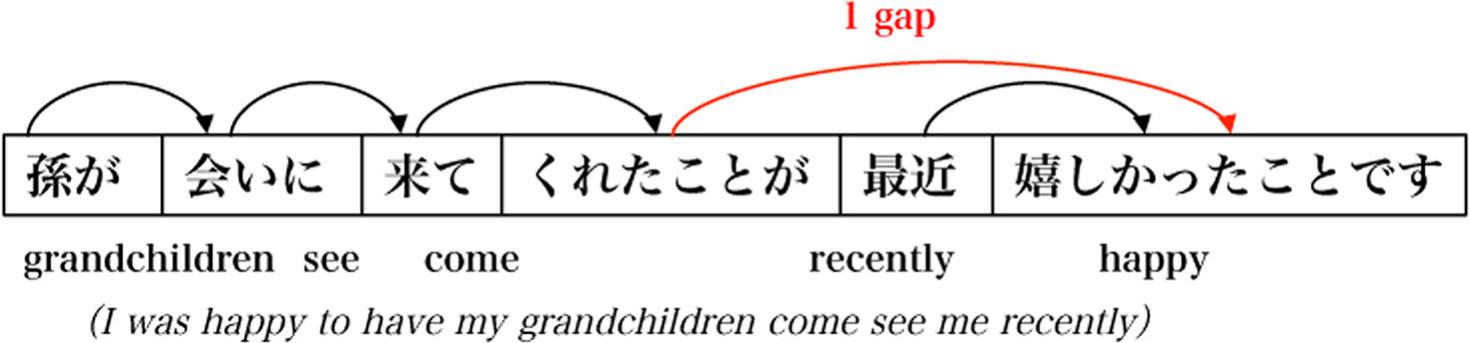
Dependency distance.

The Dependency Distance (DepD) is a metric that demonstrates the average dependency distance for each phrase in a narrative. To obtain dependency structure, we used a Japanese dependency and case structure analyzer, KNP. DepD was derived using the following equation:

$$DepD=\frac{\sum_{w\in W}DependencyDistance(w)}{\left|W\right|}$$

where W is the set of nouns and |W| is the number of words in a document. The *DependencyDistance(w)* is the number of gaps in the dependency structure (Fig 2). As it is difficult to parse a phrase with a lengthy dependency relation, this index corresponds to the difficulty of sentence comprehension.

#### (5) Yngve Score

**Fig 3:**
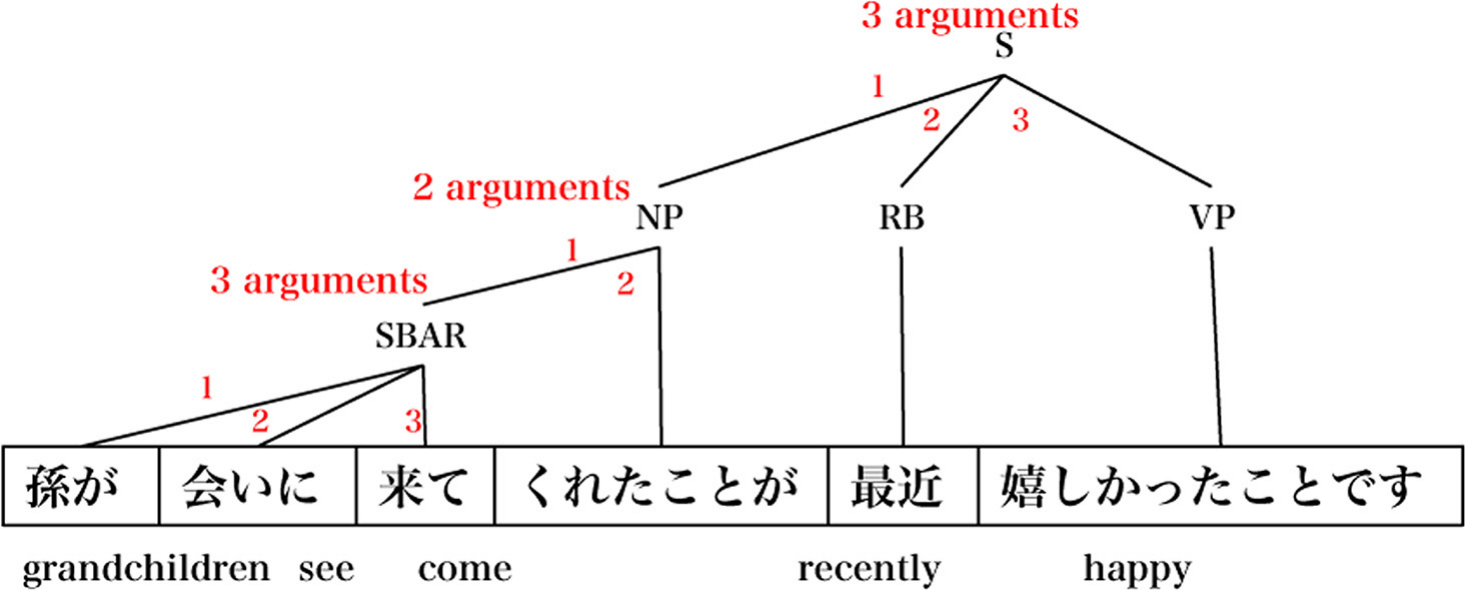
Yngve score.

The Yngve score [36] is based on parse tree shapes, where the number of branches for each node represents the number of arguments for a phrase. For this study, we have modified the Yngve score to fit the Japanese language dependency structure (Fig 3). This index corresponds to the grammatical complexity of a sentence.

$$Yngve\;score=\frac{\sum_{v\in V}No.\;of\;arguments(v)}{\left|V\right|}$$

where V is the set of verbs in a document.

### Experimental Procedure

We investigated the relationships between possible MCI and each of the language ability scores in the following 3 datasets: (1) written narrative (which was analyzed first), (2) spoken narrative (which was analyzed next), and (3) the gap in content between the spoken and written narratives. The gap in content between speaking and writing was calculated using the ratio of the language ability scores between the spoken narrative and the written narrative (i.e., speaking score/writing score), which was designated “GAP-S/W”.

A Perl program was used to examine the language abilities of the participants. We also conducted Mann–Whitney *U* tests to compare the language abilities between the Healthy and Low HDS-R Groups. Statistical significance was set at *p* ≤ .05.

## Results

The length of writing and speech varied widely among the participants. The written narratives ranged from 210 characters to 958 characters, while the transcribed spoken narratives ranged from 64 characters to 1,368 characters. The means and SDs for content length are shown in Table 4. There were no significant correlations between the length of writing and speech (*r* = .14; when n = 22, *r* must be higher than .42 to show correlation).

**Table 4:** Number of characters in the spoken and written narratives.

	Healthy	MCI
	(HDS-R score of 27 or higher)	(HDS-R score of 26 or lower)
**Mean number of characters in speech (SD)**^**a**^	462.86 (369.55)	338.75 (291.80)
**Mean number of characters in writing (SD)**	465.29 (116.35)	553.50 (210.14)

HDS-R, revised Hasegawa Dementia Scale; MCI, mild cognitive impairment; SD, standard deviation

^a^ The number of characters in speech was counted after phonetic transcription.

The differences in language ability scores for the written narratives between the Healthy Group and the Low HDS-R group are presented in Table 5. Our analysis showed no significant differences between the 2 groups in any of the scores. Although the TTR score showed the largest difference between the Healthy Group (mean: 0.51; SD: 0.04) and the Low HDS-R Group (mean: 0.48; SD: 0.03), this difference was relatively small and non-significant (*p* = .11).

**Table 5:** Writing.

	Writing (W)		
	Healthy	MCI	*p*
TTR	0.51	0.48	n.s.
PVS	1596	1254	n.s.
VL	0.51	0.53	n.s.
DepD	1.42	1.70	n.s.
Yngve	1.47	1.55	n.s.

TTR, Type-Token Ratio; PVS, Potential Vocabulary Size; VL, Vocabulary Level; DepD, Dependency Distance.

Underlined values indicate statistically significant results at *p* ≤ .05

The differences in the language ability scores for the spoken narratives between the Healthy Group and the Low HDS-R group are presented in Table 6. The PVS score showed a significant difference (*p* = .05) between the Healthy Group (mean: 541.93; SD: 409.53) and the Low HDS-R Group (mean: 2,668.38; SD: 3522.78). TTR also showed a small, albeit non-significant, difference at a level similar to that of the written narratives (*p* = .11).

**Table 6:** Speech

	Speech (S)		
	Healthy	MCI	*p*
TTR	0.43	0.53	n.s.
PVS	541	2668	n.s
VL	0.41	0.40	n.s.
DepD	2.03	2.05	n.s.
Yngve	1.60	1.52	n.s.

TTR, Type-Token Ratio; PVS, Potential Vocabulary Size; VL, Vocabulary Level; DepD, Dependency Distance.

Underlined values indicate statistically significant results at *p* ≤ .05

The differences in the language ability scores for GAP-S/W between the Healthy Group and the Low HDS-R group are presented in Table 7. The TTR score showed a significant difference (*p* = .04) between the Healthy Group (mean: 0.84; SD: 0.20) and the Low HDS-R Group (mean: 1.11; SD: 0.35). In addition, the PVS score also showed a significant difference (*p* = .01) between the Healthy Group (mean: 0.41; SD: 0.35) and the Low HDS-R Group (mean: 1.63; SD: 1.55).

**Table 7:** GAP-S/W.

	Ratio (S/W)		
	Healthy	MCI	*p*
TTR	0.84	1.11	n.s.
PVS	0.41	1.63	0.014
VL	0.82	0.78	n.s.
DepD	1.42	1.46	n.s.
Yngve	1.39	1.15	n.s.

TTR, Type-Token Ratio; PVS, Potential Vocabulary Size; VL, Vocabulary Level; DepD, Dependency Distance.

Underlined values indicate statistically significant results at *p* ≤ .05

## Discussion

Using NLP-based language ability scores, we compared the written and spoken language abilities between elderly individuals with and without suspected MCI.

### Preservation of Writing Ability in the Low HDS-R GROUP

As shown in Table 5, our findings demonstrated that there were no significant differences in the language ability scores between the healthy elderly individuals and those who may have MCI. These results suggest that writing abilities may be preserved when cognitive impairment is relatively mild. A possible reason for this observation is that the circumstances of the writing assignment did not prevent the participants from looking up words in dictionaries or receiving help from others, even though we had requested that they complete the assignment without external help. Participants in both the Healthy Group and the Low HDS-R Group had equal opportunities to look up words or ask for help from others, and these efforts may also represent writing skills. In order to reduce stress in the participants, we did not stipulate a time limit for the writing assignment, which may have given participants sufficient time to recall more words and phrases.

### Variations in Speaking Ability in the Low HDS-R GROUP

**Fig 4:**
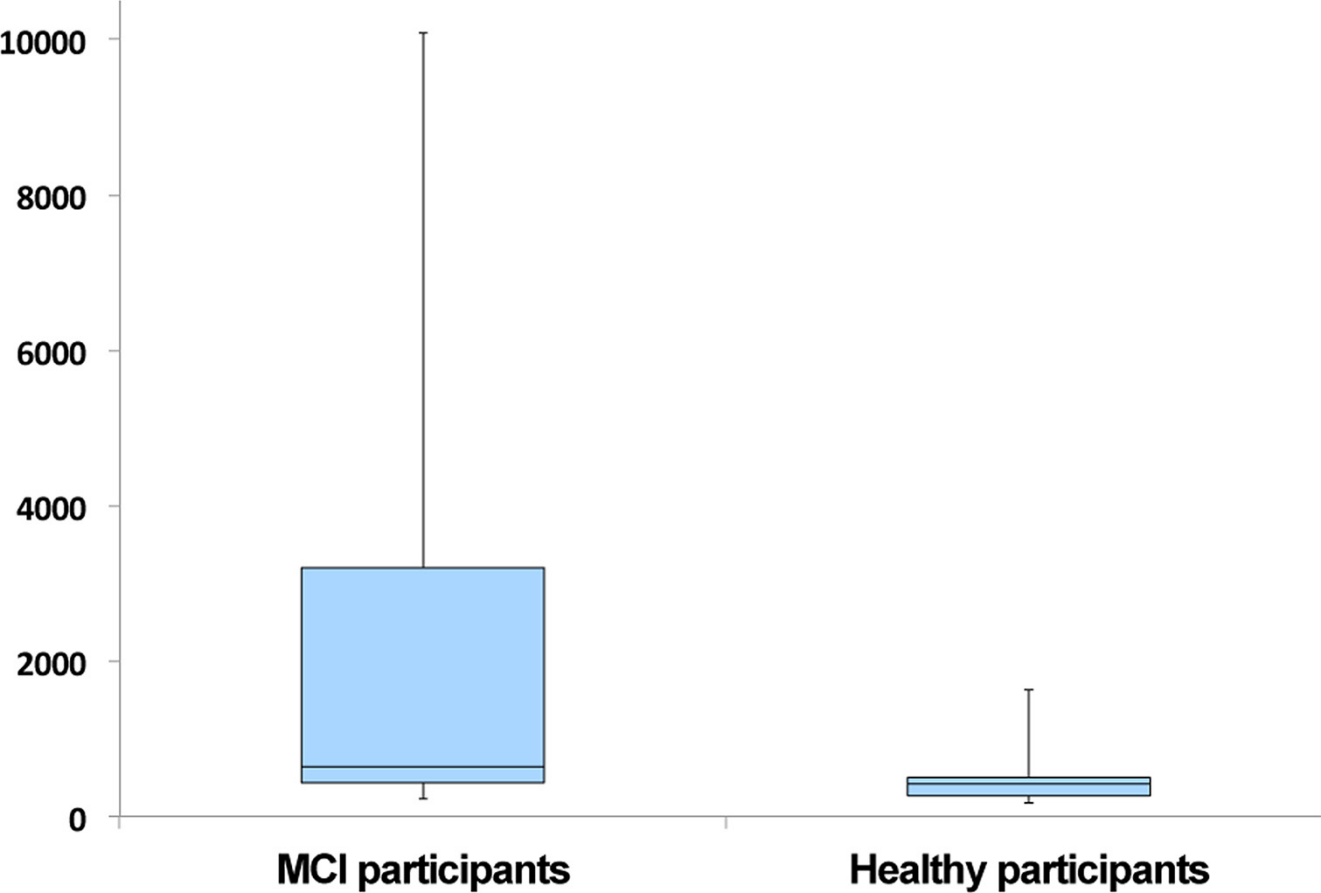
Box plots of PVS scores of MCI and Healthy participants.

As shown in Table 6, the Low HDS-R group participants had significantly higher PVS (*p* = .05) scores when compared to the healthy participants, indicating that the former had a richer vocabulary in speech than the latter. These results appear to contradict previous findings [37–39] that have reported the deterioration of language abilities in speech after the onset of dementia. However, the PVS scores in our study showed substantial variations within the Low HDS-R group (SD: 3,522.78), whereas the healthy participants’ PVS scores were more consistent (SD: 409.53). The box plots for the PVS scores are shown in Fig 4. It was also notable that participants with HDS-R scores that were lower than 22 also had very low PVS scores, whereas those who scored 25 or 26 (which may be considered the borderline between being healthy and MCI) had high PVS scores; however, those with HDS-R scores of 27 or higher (indicating no cognitive dysfunction) had PVS scores that were lower than those of the borderline HDS-R score group. This may indicate that individuals developing MCI may temporarily demonstrate an increase in PVS. A possible explanation is that patients with early-stage MCI engage in behavior to conceal their deteriorating cognition, and the underlying insecurity may actually increase their loquaciousness and active vocabulary. Follow-up studies are needed to investigate this issue further.

### Gap between Speaking and Writing in the Low HDS-R GROUP

There were more significant differences in language ability scores between the Healthy Group and the Low HDS-R group in GAP-S/W than in the written and spoken narratives alone. As shown in Table 7, the GAP-S/W scores of the Low HDS-R participants showed significantly higher TTR (*p* = .04) and PVS (*p* = .01) scores when compared with the healthy participants. Analysis of the gap between speaking and writing abilities revealed more prominent differences between the Low HDS-R participants and the healthy participants, indicating that this metric may have applications in the early detection of MCI.

### Comparisons with Related Research

Our overall findings showed significant differences between the Low HDS-R participants and healthy participants only in the TTR and PVS scores, both of which are indicative of vocabulary size. This suggests that although individuals with MCI may initially exhibit changes to vocabulary size, not many may suffer from grammatical or word difficulties. These results corroborate the findings of Lyons et al. [40].

In our results, TTR demonstrated better performance when the amount of analyzed text was relatively consistent in writing (mean: 497.36 characters; SD: 162.70), as shown in Table 5. In contrast, PVS had better performance when the amount of analyzed text had a larger degree of variation in speech (mean: 417.73 characters; SD: 348.47), as shown in Table 6. These observations may be because PVS is a more accurate indicator of vocabulary size.

Our study shows a possible correlation between MCI and vocabulary size in speech and GAP-S/W. This suggests that analyzing the speech of elderly individuals may allow the detection of very early stages of MCI. This result may appear contradictory to the previous report by Croisile et al. [41], which concluded that written texts are more reliable than oral texts in the detection of dementia. However, that report focused on patients diagnosed with AD, whereas our study examined the early stage that precedes the onset of dementia. This difference in study subjects may explain the different findings. Our results indicate that it may be possible to detect the early stages of reduced cognition before dementia onset through the analysis of spoken narratives.

These findings are the collective results of the study sample, and may represent an initial step for subsequent applications in early-stage dementia screening in individual subjects. Prompt diagnoses will enable patients to receive support or to seek proper treatment at healthcare institutions as early as possible. This study’s aim was not to develop an alternative diagnostic method that replaces the diagnoses of experienced physicians, but rather to offer assistance to both patients and physicians in consideration of the insufficiency of dementia specialists who can diagnose the growing number of dementia patients in Japan.

### Limitations

This study has several limitations. First, our study comprised 22 participants, 8 of whom had suspected MCI. As this was not a very large sample, future studies with larger sample sizes are needed to verify our findings. Next, the test that we used to examine the participants’ cognitive abilities does not distinguish between the different types of dementia, which may affect the generalizability of the results. As the characteristics associated with each type of dementia are different, there is a need for simple methods to accurately identify these conditions. In addition, we did not stipulate a time limit for the writing assignment, nor did we require the participants to write a large amount of text. Therefore, the results may be different if the participants had to write longer narratives. Further investigations are needed to shed light on this relationship and to verify the accuracy and applications of our candidate indicator.

## Conclusions

This study quantitatively analyzed the written and spoken narratives of elderly individuals with and without MCI using NLP-based techniques in order to examine the relationship between cognitive ability and language ability. The gap in content between the written and spoken narratives (PVS: *p* = .01) was significantly larger in the Low HDS-R Group. Although there is the possibility of sampling bias due to the lack of random sampling, the results indicate the basic feasibility of dementia screening using a language-based analysis, and our approach may facilitate the generation of new indicators and methods that are less susceptible to investigator-level variations.
